# Reorganization of perineuronal nets in the medial Preoptic Area during the reproductive cycle in female rats

**DOI:** 10.1038/s41598-020-62163-z

**Published:** 2020-03-25

**Authors:** Natalia Uriarte, Marcela Ferreño, Diego Méndez, Javier Nogueira

**Affiliations:** 1 Laboratorio de Neurociencias, Facultad de Ciencias, Universidad de la República, Montevideo, 11400 Uruguay; 2 Departamento de Histología y Embriología, Facultad de Medicina, Universidad de la República, Montevideo, 11800 Uruguay

**Keywords:** Cellular neuroscience, Social behaviour

## Abstract

Perineuronal nets (PNNs) are aggregations of extracellular matrix associated with specific neuronal populations in the central nervous system, suggested to play key roles in neural development, synaptogenesis and experience-dependent synaptic plasticity. Pregnancy and lactation are characterized by a dramatic increase in neuroplasticity. However, dynamic changes in the extracellular matrix associated with maternal circuits have been mostly overlooked. We analyzed the structure of PNNs in an essential nucleus of the maternal circuit, the medial preoptic area (mPOA), during the reproductive cycle of rats, using the *Wisteria floribunda* (WFA) label. PNNs associated to neurons in the mPOA start to assemble halfway through gestation and become highly organized prior to parturition, fading through the postpartum period. This high expression of PNNs during pregnancy appears to be mediated by the influence of estrogen, progesterone and prolactin, since a hormonal simulated-gestation treatment induced the expression of PNNs in ovariectomized females. We found that PNNs associated neurons in the mPOA express estrogen receptor α and progesterone receptors, supporting a putative role of reproductive hormones in the signaling mechanisms that trigger the assembly of PNNs in the mPOA. This is the first report of PNNs presence and remodeling in mPOA during adulthood induced by physiological variables.

## Introduction

The adaptation of the mammalian female brain to motherhood both prior to parturition and after the birth of the young involves structural and functional remodeling of several areas and circuitries. Most of these changes begin during gestation and the early postpartum period and continue throughout lactation when structural and functional properties dynamically adapt to the needs and demands of the young^1–4^. Although these processes constitute one of the most relevant periods of plasticity in the adult brain in natural conditions^5^, its underlying cellular and molecular mechanisms are yet poorly understood.

Among the brain regions influenced by these peripartal modifications are those directly involved in the expression of maternal behavior, as the medial Preoptic Area (mPOA) and the nucleus accumbens, as well as other areas not typically associated with the specific maternal circuit^3,6,7^.

Morphology of mPOA neurons changes during the reproductive cycle. Spine density^8^, and the number and length of basal dendritic branches increase in late-pregnant^9^ compared to non-pregnant rats. Similarly, mPOA neurons of pregnant females show an increase in somatic size compared to those of virgin females, which decreases gradually from lactation day 5 to 21^9^. Similar changes were also reported in other hypothalamic nuclei, as the supraoptic nucleus, with an increase in somatic size as well as in the number of dendrites and synaptic contacts during the postpartum period^10,11^.

Extracellular space in the central nervous system (CNS) is occupied by the extracellular matrix (ECM) synthesized by neurons and glial cells^12^. In several brain regions, the ECM adopts the shape of a relatively rigid and unique lattice-like structure, enveloping the soma and proximal dendrites of neurons^13–17^. These conspicuous components, known as perineuronal nets (PNNs), are formed by chondroitin sulfate proteoglycans as its principal molecular constituent, interacting with other ECM components including hyaluronic acid (HA), tenascin-R, and link proteins^13,15^. Early reports showed the association of these structures with specific neuronal populations, mainly GABAergic parvalbumin-expressing neurons^15,17,18^. Two additional ECM components have been described: a relative loose and homogeneous constituent mainly composed by HA that surrounds the soma, dendrites, and synapses of most neuronal types, and another one composed by the extracellular domain of plasma membrane-anchored proteins^19^.

Rather than being the inert and stable scaffold early described, the ECM has structural and dynamic properties that are involved in the regulation of different processes at the CNS as neurodevelopment^15,17,18^, neuroplasticity^20^, and regulation of neuro-glial interaction^17,21^. Notably, during early postnatal life, CNS organization is shaped by experience due to the high plasticity that characterizes the time windows known as critical periods^22^. Interestingly, ECM expression and particularly PNNs assemblage are considered decisive factors involved in the closure of such critical periods^23,24^. Concordantly, studies performed in adult individuals showed that PNNs are involved in the stabilization of synaptic contacts, limiting structural and functional plasticity^23^.

Most of the work analyzing PNNs in mammals has been performed in the cortex of male rodents with few studies addressing subcortical regions such as the hypothalamus, arcuate nucleus, amygdala and basal ganglia^25–30^. Moreover, to the best of our knowledge, only one study included female brains when analyzing the organization of the ECM in rats. However, results from female and male brains were neither compared nor reported separately^31^. As far as we know, there is no information on whether these structures could be associated with the strongly conspicuous changes that characterize reproductive processes in females.

Recent work has started to explore the role of PNNs in the regulation of learned behaviors^20,23,30^. In adult male rats, the degradation of PNNs with ABC-chondroitinase in the amygdala reactivates the extinction of preexisting memories associated with drug addiction^27^ and fear conditioning^30^. Likewise, Banerjee *et al*.^20^ showed that the dynamic regulation of molecular components expressed in the PNNs of the auditory cortex is crucial for the acquisition and consolidation of fearful memories. This evidence supports the idea that PNNs composition and organization is dynamically regulated, sustaining the plasticity processes that underly learning and memory in experimental paradigms^20^.

Despite the large amount of data showing the role of extracellular matrix in neuroplasticity regulation, its role in the basic areas of the maternal circuitry is still unknown. The link between the functional and structural changes of this circuitry throughout pregnancy and lactation, and the expression of PNNs in this area during those periods, remains to be unveiled. We hypothesized that PNNs are associated with areas within the maternal circuit and are modified during the different phases of the female reproductive cycle. In the present work, we analyzed the expression of PNNs in an essential nucleus of the maternal circuit, the medial preoptic area (mPOA), in pregnant (Gestation Day (GD) 10, GD14, GD18 and GD21) and postpartum (Lactation Day (LD) 2, LD7 and LD 22) rats as well in weaned mothers. To address the influence of reproductive hormones on PNNs assembly we compared these results with the PNNs expression in mPOA of males and virgin females on diestrous, proestrous and estrous phases of the estrous cycle as well as in ovariectomized females submitted to different hormonal treatments including estrogen, progesterone and cabergoline, a dopaminergic agonist that act as an inhibitor of prolactin secretion^32^.

Further characterization of the molecular phenotype of the neurons expressing PNNs on mPOA was achieved by performing a double labeling immunohistochemistry for WFA + estrogen receptor α (ERα) and WFA + progesterone receptor (PR).

## Results and Discussion

### Perineuronal nets change along the female reproductive cycle

A profuse WFA-positive signal for difusse ECM and PNNs was found in different brain structures, a fact previously reported^15,17,18,33^. One of the most prominent WFA-positive regions was the neocortex, showing the characteristic layered pattern related to its intrinsic laminar organization (see Supplementary Fig. S1) and a Golgi like staining pattern on a subset of neurons (see Supplementary Fig. S1)^33^. Most of the neocortical PNNs stained neurons showed the stellate morphology that characterizes the GABAergic interneuron phenotype.

Neural plasticity in adult females is strongly influenced by gonadal hormones^34,35^. The mPOA is one of the most sensitive brain regions to this endocrine factors, having the highest density of steroids receptors^36,37^. Therefore we assessed the ECM expression in this area along the female reproductive cycle. Contrary to the high PNNs expression detected in the cortex, no WFA staining was detected at the mPOA of cycling females (see Supplementary Fig. S1).

**Figure 1:**
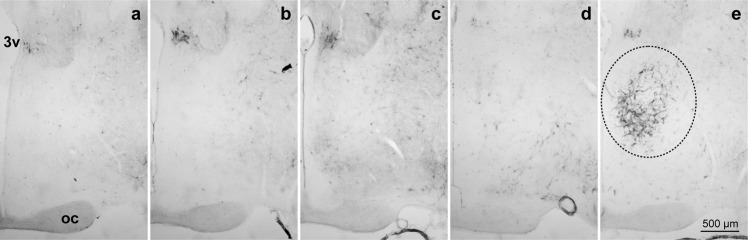
WFA staining was detected in the mPOA of pregnant rats while not present in cycling females or males. Female mPOA at (**a**) diestrous, (**b**) proestrous and (**b**) estrous, showed no detectable WFA staining. Male (**d**) mPOA showed few occasional incomplete somatic stained neurons. In contrast, pregnant females at GD 18 (**e**) showed high levels of WFA staining in the mPOA. 3 v = third ventricle. mPOA = medial preoptic area. oc = optic chiasm. Bregma –0.92 mm.

As shown in Fig. 1a–c, neither PNNs nor diffuse staining were detected in diestrus, proestrus, and estrus phases. A small set of scarce perisomatic PNNs stained neurons, together with some interneuronal diffuse signal, was detected in the mPOA of male rats (Fig. 1d). However, when WFA staining was assessed on GD18, we found a massive expression of ECM at mPOA localized predominantly in the dorsomedial nuclear region, as well as PNNs structures (Fig. 1e).

The results regarding virgin females and males are in accordance with previous studies, as Seeger *et al*.^31^ reported the absence of PNNs in the mPOA of male and non-pregnant-female rats (with no specified reproductive status). More recently, Horii-Hayashi *et al*. analyzed male mice PNNs during postnatal development excluding the mPOA since the absence of those structures in this area was assumed^28^. Our results, showing the high levels of ECM organization in pregnant rats, constitute the first report of a different pattern of ECM organization during adulthood, induced by a natural physiological condition.

In addition to its key role in the expression of parental and sexual behavior^38–40^, the mPOA is involved in neuroendocrine functions as the secretion of gonadotropin-releasing hormone, among other endocrine mediators^41^. As pointed out by Horii-Hayashi *et al*., in cycling females the mPOA would require high levels of neural plasticity, both structurally and synaptically, to adequately respond to the hormonal levels in the circulation, a fact that might explain the absence of PNNs^26^.

Thus, the high organization level of the PNNs during pregnancy, in contrast with its absence in cycling rats, could be the result of a transition from a cyclic pattern of activity to a tonic mode, where the gradually and sustained increase in pregnancy hormones levels allows the maintenance of pregnancy and its adaptive changes.

### The mPOA WFA staining is associated with a PNNs expressing neuronal subpopulation

**Figure 2:**
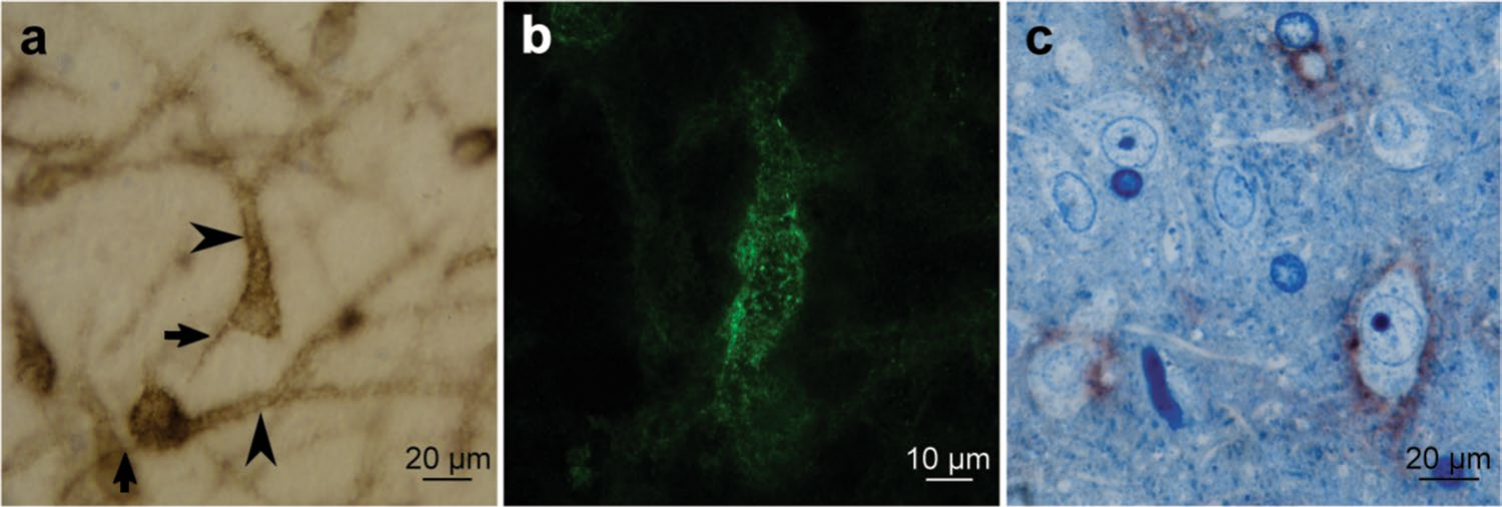
Golgi like stained cells in the mPOA of pregnant females are neurons. (**a**) Golgi like stained neurons in mPOA at GD18. Soma, dendrites (arrows), as well as axon initial segment (arrowheads) shows WFA associated DAB staining; (**b**) Confocal images z-stack of fluorescent labeled WFA preparations, showed that PNNs in the mPOA are organized in a “honeycomb” structure as described for neocortical PNNs together with a distributed punctuated pattern; (**c**) mPOA semithin sections at GD18 confirm the neuronal identity and show the external surrounding distribution of the WFA associated DAB staining. Positive surface stained cells show neuronal features, including big heterochromatic nuclei with prominent nucleolus, and abundant cytoplasm containing Nissl bodies. Interestingly, some neurons are not stained indicating that PNNs positive cells correspond to a neuronal subpopulation within this mPOA region.

High magnification images of WFA-DAB stained mPOA (Fig. 2) show that the increase in the ECM staining detected during gestation is mainly due to the appearance of PNNs expressing cells, rather than to an interneuronal diffuse component. In-depth analysis showed a Golgi like staining pattern that is consistent with neuronal cells, showing cellular processes similar to dendrites and axonal initial segments (Fig. 2a). Moreover, the detection of WFA with fluorescent coupled streptavidin showed a complex PNNs structure composed by holes, as those classically described in honeycomb-like neocortical PNNs organization^13,42^, combined with a distributed punctuated pattern (Fig. 2b). This peculiar PNNs organization requires further analysis, exploring the fine structural organization, the molecular components involved, and its relation with the synaptic contacts distribution.

To further confirm if the WFA Golgi like pattern is associated with neurons, semithin sections of previously WFA stained slices were made using toluidine blue to counterstain sections. As showed in Fig. 2c, WFA-associated DAB stained PNNs envelop neuron shaped cells that exhibit big heterochromatic nuclei with prominent nucleolus and cytoplasm containing Nissl bodies. Interestingly, we found neighbor DAB unstained cells, indicating that the expression of PNNs is associated with a subset of neurons within the regions of the mPOA here analyzed.

This neuronal subtype specificity matches the complexity and structural heterogeneity of the mPOA which contains cells expressing different neurotransmitters and neuropeptides^43,44^. Previous reports showed that 50 to 95% of neurons in the mPOA and ventral bed nucleus of the stria terminalis (vBNST) synthesize the inhibitory neurotransmitter GABA^43,45^. Interestingly, recent data showed that in maternal mice and following a pup exposure procedure, most Fos-expressing neurons in the mPOA and in the vBNST are inhibitory GABAergic neurons, while very few appear to be glutamatergic^43^. On the other hand, while PNNs could be found surrounding various cell types in different brain regions^46–48^, they mainly enwrap a subpopulation of inhibitory neurons, the fast-spiking parvalbumin-expressing (PV+) neurons in the neocortex^46,49–51^. It is then tempting to speculate that the neurons in mPOA expressing PNNs are GABAergic PV+ cells.

We can postulate that PNNs could be mainly associated with GABAergic neurons that are part of the maternal circuit. To further sustain this hypothesis, we are currently working to address the neurochemical identity of the PNNs associated neurons in the mPOA and its projection targets as well as its activations in different behavioral contexts.

**Figure 3:**
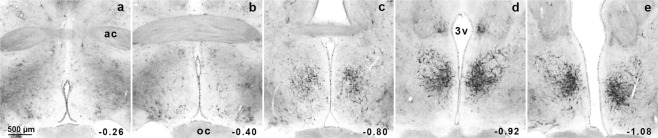
PNNs expression in the mPOA during gestation. Panels (a–e) show representative slices from anterior to posterior mPOA of GD21 female brain. PNNs were evidenced using WFA staining. Notice the appearance of PNNs expressing neurons and its antero-posterior pattern distribution, with no WFA signal in the anterior (**a**,**b**), moderate in the medial (**c**), and high in the posterior region (**d**,**e**). Numbers in each panel indicate the distance caudal to bregma in millimeters.

Despite the role of the mPOA in the establishment and maintenance of maternal behavior following parturition^5,52^, few data are available regarding whether different subregions within this area might be more relevant^43^, as shown in other behaviors^53^. For this reason, we aimed to deeply characterize the distribution of PNNs expressing neurons by performing a regional analysis of serially sectioned brains. We found that the WFA neuronal subpopulation was not homogeneously distributed along mPOA. Even more, antero-posterior analysis on GD21 showed a particular PNNs spatial distribution starting with few labeled neurons in the anterior region, increasing in medial mPOA with a maximum in the caudal portion of the nucleus (Bregma −0.92 to −1.08 mm) (Fig. 3).

How the population of PNNs expressing neurons is involved in the neural circuit responsible for the emergence of maternal behavior is still unclear. In fact, most of these neurons are located in the medial portion of the dorso-ventral mPOA axis, a location identified by Tsuneoka *et al*.^43^ following excitotoxic lesions as crucial for maternal behavior expression. Surprisingly, the medial mPOA portion is not the region where cFos expression occurs in response to pup exposure^43^. In order to understand this complex organization, it is necessary to perform a detailed anatomical study analyzing the antero-posterior distribution of PNNs together with the labeling of the different neurotransmitters involved and the cFos expression in response to maternal experience.

### The pattern of expression of PNNs in the mPOA dynamically changes throughout pregnancy

**Figure 4:**
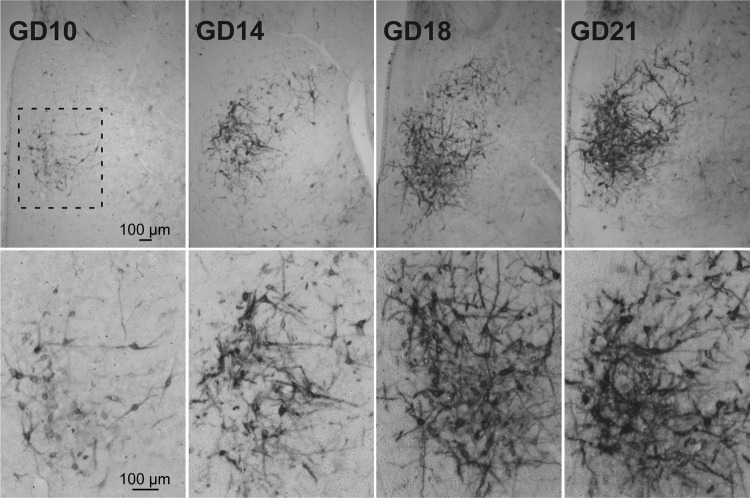
PNNs assembly during gestation in the mPOA. Upper row shows, at low magnification, the increase in PNNs expressing neurons from gestation day (GD) 10 to GD21 at the mPOA. Lower row shows, at medium magnification, neuronal shaped cells in the region enclosed by the square (GD10, upper row). Notice the increase in the number of neurons coated by PNNs as well as in the staining intensity. Note the diffuse inter neuronal staining at GD18 and 21. Bregma −1.08 mm.

To investigate the temporal pattern of PNNs organization in the mPOA during the pregnancy, we analyzed WFA reactivity on different stages of gestation (GD10, GD14, GD18, and GD21). We found that the expression of PNNs in this area was not constant during the gestation, but rather showed a gradual increase over the progression of this period, starting close to GD10. As showed in Fig. 4, on GD10 few PNNs surrounded neurons are observed and no interneuronal diffuse signal was detected. However, on GDs14 and 18 the expression increases, reaching the maximum extension and intensity of PNNs (with a small amount of interneuron diffuse staining) before parturition (GD21, Fig. 4). It is worth noting that the first PNNs expressing neurons appear in the ventromedial edge of its final distribution, progressing later towards the dorsolateral edge, ending in an eccentric ring shape (GD21, Fig. 4).

This variable pattern of the ECM expression could be attributed to the heterogeneity and complexity of the mPOA and its regions, which includes several nuclei with different subpopulations of neurons expressing specific sets of neurotransmitters^49^, regulated by several and diverse factors.

### Hormone simulated-pregnancy induces PNNs expression in the mPOA of ovariectomized rats

The dynamic changes in the expression of PNNs observed resemble the temporal profile of the hormonal levels during gestation. In rats, circulating estrogens are low for the first two weeks of gestation gradually increasing until parturition, while plasma progesterone slowly rises throughout the first two weeks of pregnancy, reaching a peak in the third week and declining abruptly before parturition. On the other hand, pituitary prolactin is secreted in two daily surges until mid-pregnancy when its secretion is inhibited by placental lactogens. This inhibition terminates towards the end of gestation with a large prepartum surge of prolactin^54–56^.

**Figure 5:**
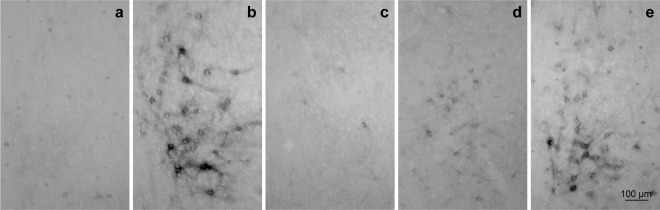
Hormone simulated-pregnancy induces PNNs expression in the mPOA of ovariectomized rats. Representative images of WFA staining in the mPOA of ovariectomized females following oil (**a**), estrogen + progesterone (**b**), estrogen (**c**), progesterone (**d**), and estrogen + progesterone + cabergoline (**e**) treatment. Notice the presence of estrogen + progesterone induced PNNs (**b**), and a lower intensity label with the addition of cabergoline (**e**). Interestingly, estrogen treatment alone does not induce PNNs expression (**c**), while progesterone treatment showed a subtle perisomatic WFA label (**d**). Bregma −1.08.

This suggests that the prolonged exposure to estrogen, progesterone and/or prolactin during this period could be related to the assembly of the ECM in the mPOA. To explore this possibility, we assessed WFA reactivity in ovariectomized (OVX) rats (a) following a hormonal simulated pregnancy treatment with estrogen and progesterone^57^; (b) treated with estrogen + progesterone + cabergoline (to inhibit prolactin secretion); and (c) to each steroid separately. In accordance with the absence of PNNs labeling in cycling females, OVX oil-treated control rats did not show any label of WFA in mPOA (Fig. 5). Interestingly, following the hormone-simulated pregnancy treatment with estrogen and progesterone, the mPOA of OVX females showed PNNs assemblies around cell bodies (Fig. 5). When OVX rats were treated only with estradiol no signal of PNNs could be detected. Progesterone administration on the other hand, induced a few perisomatic PNNs labeled neurons, suggesting a complementary effect of both gonadal hormones. As shown in Fig. 5(e), the inhibition of a putative gonadal steroid- induced prolactin secretion, due to the dopaminergic agonist cabergoline, prevented the complete expression of PNNs induced by the treatment with estrogen and progesterone (Fig. 5b).

These findings indicate that steroid hormones are capable of inducing the assemblage of the PNNs in the mPOA, and that the effects could be partially attributed to an increase in the secretion of prolactin. Interestingly, although the main gonadal steroid hormones of both the estrous cycle and pregnancy are the same, the changes in the assembly of the PNNs were only observed during the pregnancy or following hormone simulated-pregnancy treatment and not during the estrous cycle. Taken together these results show that this structural plasticity is the consequence of the high and sustained hormonal levels observed during the gestation period and suggest that this phenomenon is specific of this reproductive cycle stage.

On the other hand, it is worth noting that the expression of PNNs in the mPOA was higher in pregnant rats than in hormonal-treated OVX females, suggesting that prolactin and possibly other endocrine factors present during gestation (i.e. placental lactogens, growth factors, chorionic gonadotropin) could be involved in the full expression of PNNs seen in the mPOA of pregnant rats before parturition^58^. Moreover, this difference could be explained by the fact that circulating levels of gonadal steroids induced by the pharmacological treatment differed from the natural variations of the hormonal milieu of gestation.

Recently, Fang *et al*. reported that the optogenetic activation of mPOA GABAergic neurons that express ERα and project to the ventral tegmental area strongly inhibits non-dopaminergic cells in this area and drive pup retrieval behavior in maternal mice (Fang *et al*.^63^). It is also known that estrogen can increase GABA release and reuptake as well as GABA A-receptor expression in the mPOA^59,60^, and that steroid manipulation modulates mRNA levels of glutamic acid decarboxylase (GAD) in this area^61,62^.

Despite the many possible steroid-induced neuronal phenotype alterations, given the central role of GABAergic neurons in maternal behavior^45,63^, its higher number and its association with extracellular matrix components in other regions, we hypothesized that mPOA-PNN+ neurons are GABAergic projecting neurons that express steroid receptors. Exploring this hypothesis will require immunological detection of GAD and ERα and PR, combined with retrograde labeling experiments. In a first attempt to characterize this neuronal phenotype we performed a double-label immunohistochemistry to detect ERα and WFA and PR and WFA in the mPOA of GD21rats.

### PNNs associated neurons in the mPOA express estrogen receptor α and progesterone receptors

**Figure 6:**
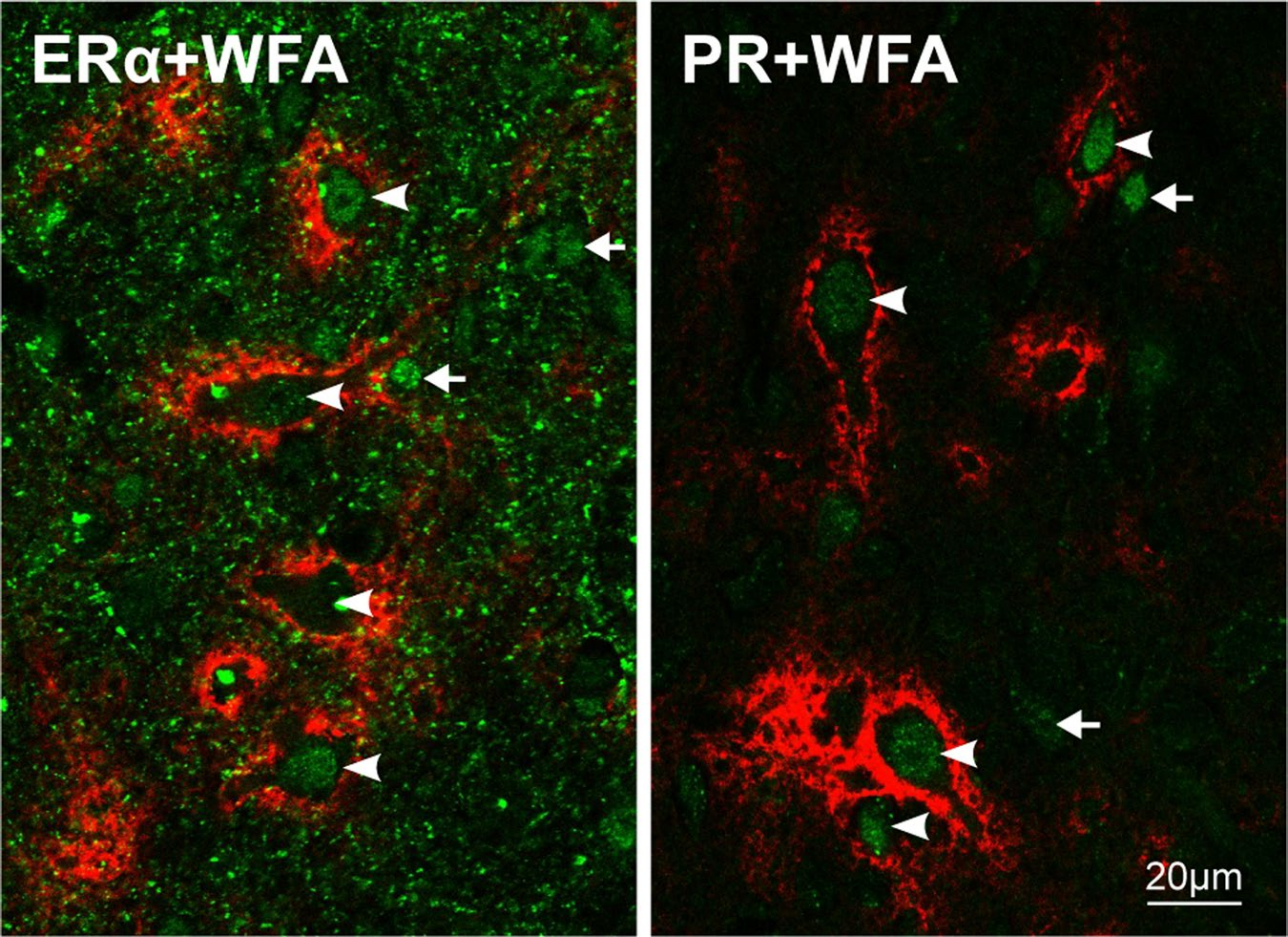
PNNs associated neurons express estrogen receptor alpha and progesterone receptor. Double labeling using WFA and anti-estrogen-receptor-alpha antibody (ERα + WFA, left) or WFA and anti-progesterone-receptor antibody (PR + WFA, right). In both cases PNN-WFA label was tagged using streptavidin-594 (red), while hormone receptors were tagged using secondary antibodies conjugated with alexafluor-488 (green). In the left panel notice the presence of cell nucleus label for ERα and its variability between different PNNs expressing neurons (arrowheads). Positive nuclei belonging to PNNs negative cells (arrows) are also evident. Interestingly, there is a conspicuous doted label in the neuropilic region that is absent in neocortical neuropilic region (Supplementary Fig. 2). Note the presence of PR label nuclei in PNNs associated neurons in the right panel. PR label intensity is less variable compared to ERα label (left panel) and lacks the neuropilic dotted component. A population of PR labeled nucleus colocalizes with PNNs negative cells (arrows).

As shown in Fig. 6, cell nuclei of PNNs associated neurons show different intensities of ERα label, suggesting variations in protein expression levels (Fig. 6-left, arrowheads) which are also evident in the nuclei of PNNs negative cells (Fig. 6-left, arrow). Additionally, we observed an abundant dotted neuropilic label probably associated to neuronal or astrocytic membrane processes^64,65^. This neuropilic label was not found in the neocortex where a conspicuous nuclear label was clearly detected (Supplementary Fig. S2).

PNNs associated neurons also express the PR label localized on the cell nucleus, however, neither mPOA neuropilic nor nuclear neocortical label was detected. Interestingly, there was a positive PR nuclear label in the PNNs negative cells (Fig. 6-left, arrow), lacking the variability in intensity observed for the ERα label (Fig. 6-left, arrowheads). As this profile of expression was observed at GD21, the analysis of temporal dynamic of the expression of both receptors along the gestation period is of great interest.

### The PNNs expression in the mPOA persists after parturition, gradually fading throughout lactation

**Figure 7:**
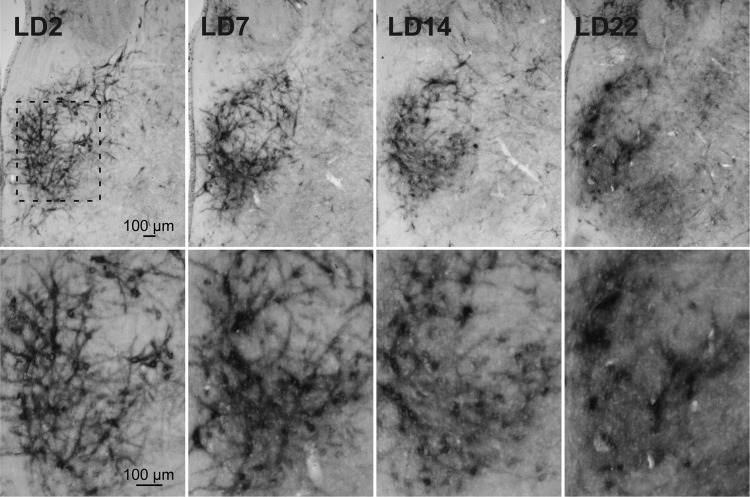
PNNs disassembly during lactation in the mPOA. The WFA expression pattern, as well as the reactivity level, gradually declined from the second day of lactation (LD2) on, undergoing a gradual remodeling through the postpartum period: a transient increase halfway through the lactation period is followed by a decrease towards LD14, resulting in a very diffuse label at postpartum day 22. Medium magnification images (lower row) show that PNNs structure start fading at LD7 with the loosing of dendritic staining, showing an increase in the remanent perisomatic diffuse staining from LD7 to LD22. Bregma −1.08 mm.

In order to advance in the characterization of the possible role of mPOA PNNs in the establishment and maintenance of maternal behavior and lactation, we aimed at understanding if the high organization of the ECM persists after the birth of the young. Following parturition, the densely packed PNNs observed in mPOA during late gestation begin to fade. As shown in Fig. 7, PNNs are present after parturition (LD2) but with a decreased expression intensity. After that, PNNs undergoe a complex dynamic degradation process along lactation. The WFA expression pattern as well as the reactivity level change gradually, declining from the second day of lactation (LD2), transiently increasing halfway through lactation (Fig. 7, LD7) and resulting in a very diffuse label at the time of postpartum day 22-23 (Fig. 7, LD22). The transient increase during lactation (LD7) seems to be due to an increase in the diffuse interneuronal extracellular matrix component, rather than an increase of PNNs expressing neurons, explaining how global WFA staining increase occurs even when PNNs are fading. By the end of the lactation period (PPD 22) only a few perisomatic PNNs positive cells are still present with a surrounding interneuronal diffuse staining. These results suggest a complex PNNs degradation process probably involving the action of matrix metalloproteinases^66^, an underlying mechanism for a programmed PNNs degradation, or a passive mechanism of disassembly combined with transient synthesis events.

To further explore the possible role of WFA stained neurons, it would be interesting to address if the early gene expression in mPOA neurons of females exposed to maternal experience is colocalized within the PNNs+ neuronal population here described.

**Figure 8:**
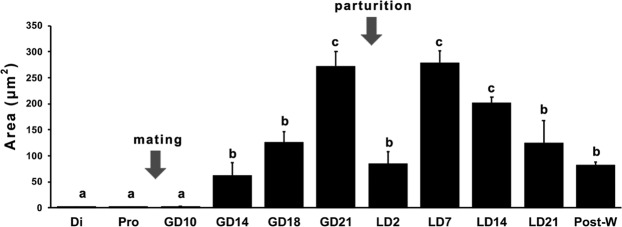
The expression of PNNs in the mPOA changes throughout the female reproductive cycle. The quantification of WFA staining waxes and wanes along the female cycle. An increase in the area occupied by the PNNs along gestation reaches a maximum immediately before parturition (GD21) followed by a reduction of staining after parturition (LD2) with a subsequent increase towards a maximum (LD7), and a final reduction of WFA staining at the end of lactation period (LD22, Fig. 8). Data are expressed as means ± SEM. Different letters indicate significant differences between groups (p < 0.05, Tukey post hoc test).

The quantification of WFA staining confirmed the qualitative analysis of PNNs expression throughout the female reproductive period, showing two different processes: (1) the increase in the area occupied by the PNNs staining in the mPOA along gestation reaching a maximum immediately before parturition (GD21), and (2) a complex dynamic in which there is an immediate reduction of staining after parturition (LD2) followed by a transient increase (LD7), and a final reduction of WFA staining through the end of lactation period (LD22, Fig. 8).

Interestingly, a trace of extracellular matrix expression on late postpartum remains up two weeks after weaning (Fig. 8, Post-W), suggesting that once a female undergoes a reproductive cycle a molecular label is generated. How these remanent extracellular components could facilitate subsequent reproductive structural and physiological changes is an interesting question to be answered.

The complexity of PNNs dynamics observed during gestation and the postpartum period suggests a fine regulatory role of the extracellular matrix on neural circuit properties. Experimental evidence points toward a profound remodeling of neuronal morphology and synaptic connections regulated by gonadal steroids^9^. On the other hand, the hormonal changes taking place during pregnancy initiate complex behavioral changes that promote maternal care of the offspring after birth. We also know that maternal behavior dynamically changes along the postpartum period, adjusting to the physiological needs of the pups^1,67^. This adaptation has been attributed to functional modifications in the maternal circuitry, mainly the mPOA, during lactation^1,68^. Although the mechanisms by which this adaptation takes place are not known, a role of the sensory stimulation provided by the pups as well as endocrine factors has been postulated^69^.

We hypothesize that if the high organization of PNNs before parturition is related to the stabilization of the maternal circuitry, the waning in its expression observed during lactation could be related to the dynamically changing expression of the maternal behavior characteristic of this period.

The phenomenon here described opens a new opportunity to explore the cellular and molecular mechanisms involved in PNNs assembly and disassembly during adulthood, raising many interesting questions: What are the cellular components involved in the secretion of the extracellular matrix? Which are the molecules responsible for catalyzing the PNNs assemblage around specific neuron populations and, which signals are driving this process? What is the role of metalloproteinases? What is the relation between PNNs and the functional role of mPOA during maternal behavior?

## Methods

### Animals and housing

Wistar rats (*Rattus norvegicus*) of both sexes three to four-month-old were used. Animals were housed under controlled temperature (22 ± 1 °C) and humidity (65%) in a 12 h light-dark cycle (lights on at 0400 h) with free access to food and water. All animals were housed in groups of three to four per cage (48 × 33 × 16 cm), except lactating females or mating couples. Mating was achieved by placing a receptive female rat with a sexually active male overnight and that day was considered as GD 0.

Lactating females were individually housed at pregnancy day 21. Following the parturition they were maintained in individual cages with their litters (adjusted to four male and four females on the delivery day). Animal care and experimental procedures were approved by the Institutional Animal Care and Use Committee of the Facultad de Ciencias, Universidad de la República (reference number: 240011-001541-17) and were in accordance with Uruguayan Law N°18,611 for the Care and Use of Laboratory Animals.

### Experimental groups

Experimental groups were designed to reveal the pattern of expression of the PNNs during the different phases of the reproductive cycle:

Virgin females at late proestrus (n = 6), estrus (n = 6) and diestrus (n = 6); pregnant females at gestation day (GD) 10 (n = 4), GD14 females (n = 5), GD18 females (n = 5) and GD21 females (n = 7); postpartum females on lactation day (LD) 2 (n = 7), LD7 (n = 6); LD14 (n = 4), LD 22 (n = 6) and two weeks following weaning (Post-W, n = 4). An additional group of males (n = 5) was included.

To determine whether reproductive hormones were responsible for the high organization of PNNs found in GD21 females we compared the expression of PNNs in the following groups: ovariectomized (OVX) vehicle-treated (n = 3), OVX estrogen + progesterone treated (n = 3), OVX estrogen-treated (n = 3), OVX progesterone treated (n = 3) and OVX estrogen + progesterone + cabergoline treated (n = 3) females.

### Estrous cycle assessment

Estrous cyclicity was monitored by daily vaginal smears (samples taken between 09:00 and 11:00 h). Four stages were established according to the different vaginal cytology: (1) late proestrus (round nucleated cells and cornified cells), (2) estrus (cornified cells), (3) metestrus (round nucleated and cornified cells and leukocytes) and (4) diestrus (predominance of leukocytes). Females were included after showing at least two regular cycles.

### Ovariectomies and hormonal treatments

Females were anesthetized with 2.0 ml/kg of a solution that contained ketamine HCl (75.0 mg/ml), xylazine (7.5 mg/ml) and acepromazine maleate (1.5 mg/ml) and ovariectomized through a ventral incision. Following a recovery period of two weeks, they were divided into two groups receiving either hormonal or vehicle (corn oil) treatments via subcutaneous injection (between 8:00 and 10:00 h for 21 days). The OVX vehicle-treated group was administered 0.2 ml of corn oil daily for 16 days and then 0.1 ml for 4 days. The OVX hormone-treated groups were treated as follow: OVX estrogen + progesterone group received a low dose of estradiol benzoate (EB, 2.5 μg) combined with a high dose of progesterone (4 mg) dissolved in 0.2 ml corn oil daily for 16 days and on days 17–21 received a high dose of EB (50 μg) dissolved in 0.1 ml of corn oil. The OVX estrogen group received a low dose of EB (2.5 μg) dissolved in 0.1 ml corn oil daily for 16 days and on days 17–21 received a high dose of EB (50 μg) dissolved in 0.1 ml of corn oil. The OVX progesterone-treated group received a high dose of progesterone (4 mg) dissolved in 0.2 ml corn oil daily for 16 days and on days 17–21 received 0.1 ml of corn oil. The OVX estrogen + progesterone + cabergoline group received a low dose of estradiol benzoate (EB, 2.5 μg) combined with a high dose of progesterone (4 mg) dissolved in 0.2 ml corn oil daily for 16 days and on days 17–21 received a high dose of EB (50 μg) dissolved in 0.1 ml of corn oil and 20 μg of cabergoline (p.o. 0.4 ml) every other day from days 1 to 21.

### Tissue preparation for immunohistochemical studies

Animals were anesthetized with sodium thiopental (100 mg/kg, i.p.) and transcardially perfused with heparinized phosphate buffer saline (PBS), followed by 4% paraformaldehyde (PFA) in 0.1 M phosphate buffer (PB) (pH 7.4). Brains were removed and post-fixed with the same fixative overnight, then cryoprotected with 15% followed by 30% sucrose (in 0.1 M PBS) until they sank, before being stored at −80 °C. Coronal sections (40 µm thick) were obtained using a Leica CM1850 UV cryostat according to the Paxinos Stereotaxic Atlas^70^. Sections were collected and stored in a cryoprotective solution (30% glycerol, 30% ethylene glycol in PB) at −20 °C until use. Slices were rinsed three times PB (pH 7.4) to remove cryoprotective solution.

For WFA staining slices were processed according to the following protocol: (1) blockade of the endogenous peroxidase with 45% ethanol, 0.3% H_2_O_2_ in PB for 20 min at room temperature; (2) three times rinse for 10 min in PB; (3) blocking solution containing 3% bovine serum albumina (BSA) + 0.2% Triton X100 in PB (PBT 0.2%), pH 7.4, for 30 min; (4) incubation with 2 mg/ml of biotinylated *Wisteria floribunda* lectin (Sigma L1516, USA) diluted at 1/500 in blocking solution (200 µl per slide in 24-well plates) at 4 °C under agitation; (5) three times rinse of 10 min in PBT 0.2%; (6) incubation with avidin-biotin-peroxidase complex (VECTASTAIN ABC) in PBT 0.2% during 2 hours at room temperature; (7) three times rinse of 10 min in PB; (8) peroxidase reaction was performed using DAB SigmaFast kit with metal enhancer during 50 seconds, and the reaction was stopped by rinsing slices with PB three times; (9) sections were mounted on slides, dried at 37 °C and cover slipped using synthetic Canada balsam.

For PNNs fluorescent labeling, endogenous peroxidase blockade step was suppressed and WFA was detected using Streptavidin AlexaFluor 488 conjugated (1/500 diluted) for 2 hours at room temperature.

For double labeling immunohistochemistry (ERα + WFA and PR + WFA), 30 µm thick slices containing the mPOA region were processed according to the following procedure: (1) three times rinse of 10 min in PBT 0.1%; (2) 30 min incubation in blocking solution containing 3% BSA in PBT 0.1%, (3) incubation with 2 mg/ml of biotinylated *Wisteria floribunda* lectin (Sigma L1516, USA) diluted by 1/500 and primary antibody, in blocking solution (200 µl per slide in 24-well plates) at 4 °C under agitation during 48 hours; (4) three times rinse of 10 min in PBT 0.1%; (5) blocking solution containing streptavidin AlexaFluor 488 conjugated (1/500 diluted) and secondary antibody AlexaFluor 594 conjugated (1/500 diluted), at room temperature; (6) three times rinse of 10 min in PB. Anti-Estrogen Receptor α antibody (Millipore-Sigma 06-935, USA) was diluted 1/200 and detected with anti-rabbit secondary antibody. Anti-PR antibody (Invitrogen-Zymed 18-0172, USA) was diluted 1/200 and detected with anti-mouse secondary antibody.

Fluorescent stained sections were mounted on slides and cover slipped using glycerol 80% in PB.

### WFA stained semithin sections

Females (n = 2) at GD18 were transcardially perfused with PBS during 30 seconds followed by 300 ml of 4% PFA + 1% Glutaraldehyde. Brains were removed, postfixed during 2 hours and cut at 40 µm using a Leika (VT1000 S) vibratome. Slices containing mPOA were processed for WFA-DAB staining as described before and dehydrated using ascending ethanol concentrations (50%, 75%, 95% and 100%) ending with anhydrous acetone before embedding with araldite resin (Durcupan, Sigma). Semithin sections (1 µm) were obtained using an RMC Boeckeler ultramicrotome (PowerTome X) and stained with 1% Boraxic Methylene Blue (BMB).

### Image acquisition and PNNs signal quantification

Figure images were taken using a Nikon Eclipse E4000 microscope coupled to a Micrometrics 319CU CMOS 3.2 Megapixel Camera. Fluorescent labeled WFA preparations images were obtained using a spectral confocal microscopy (Leica TCS SP5 II).

For quantification, images were acquired using Nikon SMZ1000 binocular microscope (3x, NA 0.3) and analyzed using ImageJ software (NIH, https://imagej.nih.gov/ij/). Images were transformed to 8bits in gray scale for quantification purposes and transformed from pixels to micrometers. Regions of interest (ROI) were defined according to the maximum extension area of PNNs positive staining inside the mPOA at GD21 in one slice per brain (−0.92 ~ −1.08 mm Bregma), using a maximum entropy threshold protocol (Image-J). The settled area of 802 μm2 was applied to all images in mPOA region bilaterally. PNNs density was calculated as the mean bilateral PNNs signal density inside the ROI. Statistical analysis was made by two-way ANOVA followed by Tukey post hoc test for independent measures (statistical significance, p = 0.05).

## Supplementary information

Supplementary figures.

## Data Availability

The datasets generated during the current study are available from the corresponding author on reasonable request.
