# Supplementary material for: Reorganization of perineuronal nets in the medial Preoptic Area during the reproductive cycle in female rats
Source: Sci Rep. 2020 Mar 25;10:5479. doi: 10.1038/s41598-020-62163-z (PMC7096482; doi:10.1038/s41598-020-62163-z)
Supplement: Supplementary file 1 — Supplementary figures. [file 41598_2020_62163_MOESM1_ESM.pdf]

Reorganization of perineuronal nets in the medial Preoptic Area during the reproductive cycle in female rats

Natalia Uriarte, Marcela Ferreño, Diego Méndez, Javier Nogueira

Supplementary Figure 1

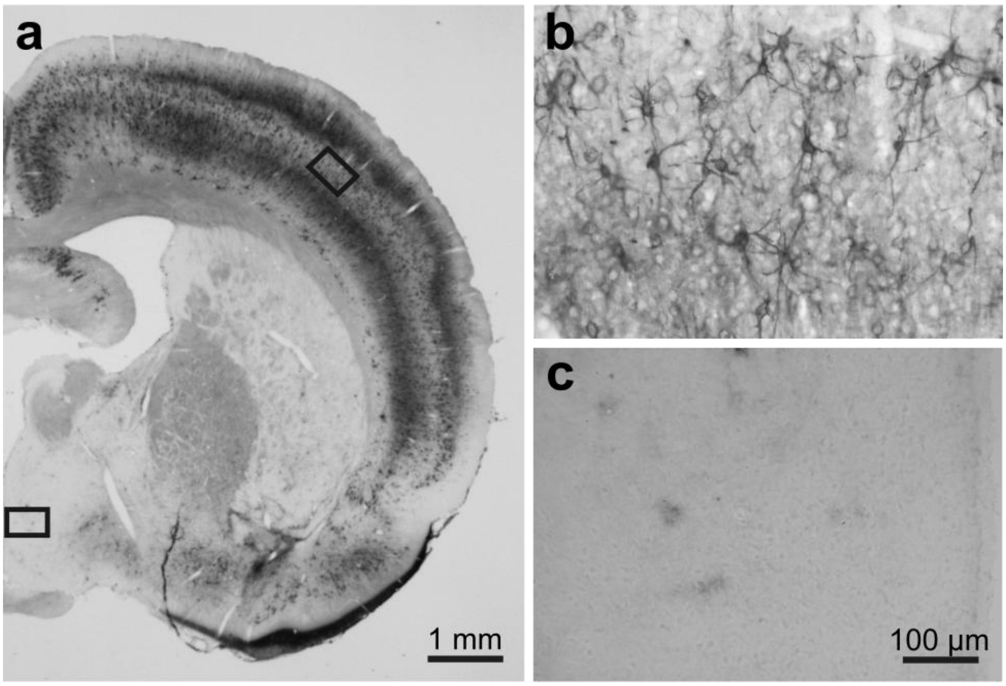

Figure 1. WFA staining in a diestrous female brain. a) The topographic image of a brain slice shows the heterogeneous distribution of the extracellular matrix along different brain structures. It is worth noting the laminar pattern in the cortex and the absence of staining in the mPOA region. b) A medium magnification image of the cortical region enclosed in the black rectangle in “a” (cortical region, upper rectangle) shows two different structures labeled with WFA a diffuse interneuronal component and a neuronal shaped component (the PNNs). c) Medium magnification image of the mPOA region enclosed in the black rectangle in “a” (lower rectangle) shows the absence of WFA staining. The few positive structures present here are neither the diffuse nor the neuronal shaped stainings shown in “b”.
Bregma -1.08 mm.

Supplementary Figure 2

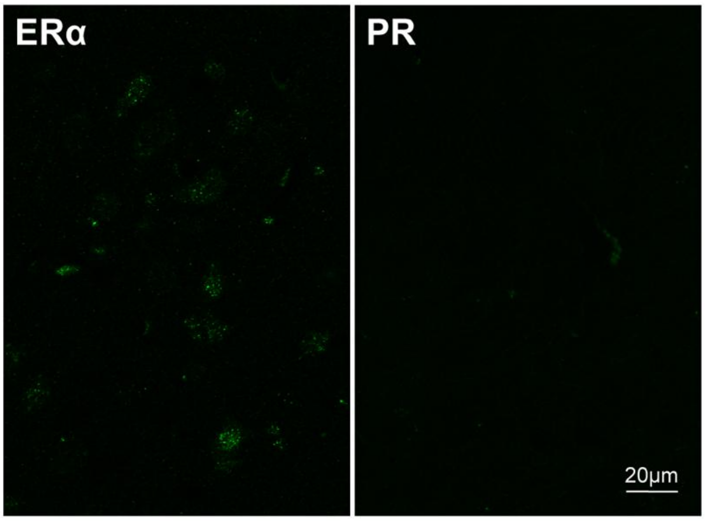

Figure 2. Neocortical expression of estrogen receptor alpha and progesterone receptor. Both pictures correspond to representative neocortical regions. On the left, immunohistochemistry staining of estrogen receptor alpha (ERα) showing a nuclear shaped and a somatic shaped label, lacking the neuropilic dotted component observed in the medial preoptic area. The right panel shows the absence of progesterone receptor (PR) staining as observed in all the neocortical regions analyzed in this study.
